# Correlation between cognitive ability and educational attainment weakens over birth cohorts

**DOI:** 10.1038/s41598-023-44605-6

**Published:** 2023-10-18

**Authors:** Arno Van Hootegem, Ole Røgeberg, Bernt Bratsberg, Torkild Hovde Lyngstad

**Affiliations:** 1 Department of Sociology and Human Geography, University of Oslo, 0349 Oslo, Norway; 2 Ragnar Frisch Centre for Economic Research, 0349 Oslo, Norway

**Keywords:** Psychology, Human behaviour

## Abstract

Educational attainment is a key indicator of status and opportunity in meritocratic societies. However, it is unclear how educational expansion has affected the link between cognitive abilities and educational attainment. As a result, this study examines the correlation between cognitive ability and educational attainment across male birth cohorts in Norway. Utilizing administrative register data covering more than four decades, we investigate multiple measures of educational attainment and their connection to cognitive abilities. Our findings reveal a consistent decline in the correlation over time. These findings question the assumed shift towards meritocracy in educational systems and highlight a more complex relationship between cognitive ability and educational attainment.

## Introduction

An established hypothesis holds that broadened societal access to educational opportunities reduces the influence of family background and increases intergenerational mobility by meritocratically lifting talented youths out of their origin class^1^. The educational system has been seen as a “stairway to opportunity” open to all, with educational credentials increasingly viewed as an indicator of skill sets and future productivity^2^.

According to these meritocratic narratives, educational opportunities are increasingly allocated on the basis of individual ability and effort rather than parental wealth and resources^3,4^. If this were so, we would expect strong predictors of educational performance, such as cognitive capabilities, to increasingly align with educational attainment. Cognitive ability does not strictly equate with merit, talent, or natural endowments, but it is a relatively stable individual-level trait that predicts academic potential, which implies that it should be increasingly aligned with educational attainment as social programs have helped level the socioeconomic playing field. This implication has, to our knowledge, not been examined systematically.

Intelligence or cognitive ability is predictive of socio-economic success later in life^5–7^. Within-individuals, cognitive ability seems to become more predictive across age for earnings and occupations, but not necessarily for education^6^. Evidence on temporal change in society at large is nevertheless sparse and hard to parse, with comparisons of samples collected at different time points indicating both stability, declining and increasing correlations^6–9^. A systematic and population-based comparison of this correlation across birth cohorts has not, to our knowledge, been conducted.

To remedy this, we examine the temporal trends in the cognitive ability-educational attainment link, calculating how cognitive ability correlates with educational attainment across four decades of Norwegian male birth cohorts (1950–1991). The Norwegian case is of particular interest, as our sample covers a period of rapid and substantial democratization of education as the Norwegian welfare state expanded after the second world war. Social differences were to be reduced through a publicly funded, broadly accessible educational system, which remains even today relatively free of privatization and a market orientation^10^. Compulsory schooling was increased from 7 to 9 years in 1969, universal rights to higher secondary education for those aged 16 and 19 came in 1994, and the school starting age was reduced from 7 to 6 in 1997^11^. Moreover, the state offer stipends and loans to anyone who enrolls in tertiary education, and credits and degrees are easily transferable across tertiary institutions.

Our analyses use Norwegian administrative register data with full population coverage and link scores from a high-quality cognitive ability test used at military conscription for men to different measures of individual educational qualifications. Across multiple methods and measures we find strong negative trends, with weakening correlations between educational attainment and cognitive ability. Correlations nevertheless remain relatively strong, pointing to cognitive ability as an important factor related to educational attainment. The loosening connection between cognitive ability and education over time nevertheless has major implications for sorting and rewards in many social domains.

## Results

**Figure 1:**
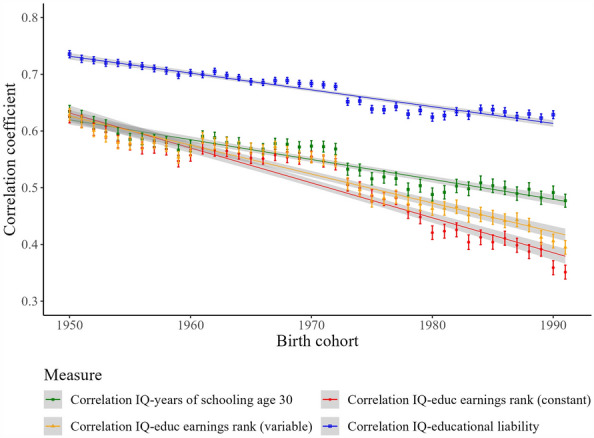
Correlation between cognitive ability and different educational operationalizations across birth cohorts (95% confidence intervals of trend lines in grey).

Figure 1 displays the correlation between cognitive ability and four different educational attainment measures calculated for each birth cohort 1950–1991. The correlation for years of education at age of 30 (in green) starts out above 0.63 for the oldest birth cohorts, but declines over cohorts ending with a correlation under 0.50 for the cohorts born after 1980. The positive bivariate relationship between cognitive ability and schooling remains, but flattens out after the 1970’s and decreases over time.

The series of correlations between intelligence and constant educational earnings ranks (i.e., earnings rank within educational degrees; shown in red) show a decline, from more than 0.60 for individuals born in the 1950’s to roughly 0.35 after 1990. This indicates a near-halving of the strength of the relationship between cognitive ability and educational attainment over one generation.

We also allow the educational earnings rank to vary across birth cohorts (orange series). After 1970 a stronger downward trend is seen, which arrives at a correlation of around 0.40 for the youngest cohorts. This indicates that the correlation with cognitive ability declines even more when we adjust education ranks by their changing economic rewards over time.

Educational attainment is a discrete outcome variable that does not conform to a normal distribution, but it can be modeled as a discretized version of an unobserved (latent) educational liability that captures all factors influencing educational attainment. Similarly, we can view a stanine score of e.g., 8 as a discretization of an underlying continuous ability scale. Polychoric correlations estimate the correlation between these latent constructs and are shown in purple. These are consistently higher, but show the same pattern with a long term secular decline. While the correlation starts slightly under 0.75 for the cohort born in 1950, it declines steadily to just above 0.65 in more recent cohorts.

A series of supplementary analyses provide further robustness checks. One concern is reverse causality, as school attendance has well-documented—albeit modest—effects on cognitive ability test scores^12,13^. To avoid picking up reverse causality, Figure S3 (in the Supplement) displays the correlations of conscription test scores with *post-test* educational achievement—i.e., using only variation in educational achievements observed after age 19. The figure shows the same downward trend as the main analysis. A second concern is that high-SES families, recognizing the increasing importance of education, may be working harder than before to push lower-ability children into education, weakening the ability-attainment gradient. To assess this, we estimate the regression slope of cognitive ability on educational attainment while controlling for parental SES (here fathers’ earning ranks), plotting the ability coefficient for each birth cohort separately. While the estimates are noisier and the trends fluctuate more, the overall pattern of decline remains clear (see Figure S4 in the Supplement).

## Discussion

Across multiple measures of educational attainment, we find a steady long-term decline in the correlation of cognitive ability scores from conscription testing and educational attainment for males in Norway. The correlation remains moderate to strong in recent cohorts and cognitive ability remains coupled to educational attainment, but the clear trend indicates that educational attainment is weakening over time as a signal of cognitive ability. This observation together with the finding that we already find a very high correlation between educational attainment and cognitive ability for birth cohorts prior to educational reforms and the democratization of education, goes counter to the hypothesis that educational attainment increasingly aligns with individual level ability as educational opportunities are broadened. Essentially, it questions the presumed evolution from ascription to achievement as the guiding principle of success in post-industrial societies^14^. Ability scores come from tests at age 18–19, which has been shown to increase the correlation between intelligence and education compared to when cognitive ability is measured at an earlier age^6^. Yet, as this age of measurement was consistent across years, it should not impact the trend in the correlations.

Norway is arguably one of the contemporary societies where it is easiest to obtain higher levels of education. From a less developed educational system, school reforms in the 1960s increased compulsory schooling for everyone in Norway^12^. Since 1994, everyone has a legal right to a place in a high school program irrespective of their past performance. The tertiary education system is relatively open and universal financial aid is provided by the government. The heritability of educational attainment has been found relatively high in Norway and similar countries^1^. Hence, this is a context where we would expect a strong correlation between cognitive ability and educational attainment to emerge, reflecting a shift towards meritocratic hierarchies following from the expansion of mass education.

The Norwegian data indicates the opposite of the expected pattern: educational attainment is over time becoming weaker as a signal of the cognitive abilities. Although this can mean different things, it has implications for the interpretation and valuation of educational attainment as an expression of an underlying trait. As cognitive ability does not equate with merit, educational attainment could still potentially be a strongly meritocratic signal of some broader set of traits, but this bundle may have become increasingly weighted towards non-cognitive factors such as motivation, perseverance, and emotional intelligence, perhaps as a result of changing demands in the labor market^15–18^. Others nevertheless argue that the role of non-cognitive skills, such as personality, in predicting socio-economic success has been overstated and does not remotely match the predictive power or cognitive capability^19,20^.

An alternative, potentially more plausible explanation for our findings, is the nature of the changing educational and labor market. Education might have become substantially less selective, as educational expansions may have made it generally easier to attain longer education, regardless of cognitive ability. This was coupled with a diversification of educational fields, whereby occupations that previously did not require higher education, and that may require other abilities than cognitive skills, might increasingly require degrees. The increased decoupling of educational attainment and cognitive ability could also happen if new educational tracks emphasize practical skills and less abstract curriculums that make cognitive ability less important. This is especially relevant in Norway, where vocationally oriented tertiary institutions are offering a wide range of educational programs and are acquiring an important status next to universities^21^. They are nevertheless still mainly attended by students with lower average grades and they have lower rejection rates, which could make the achievement of high educational attainment less dependent on cognitive ability^22^. Drawing on signaling theory^23^, educational attainment may also have become a noisier signal over time if the cost required to complete higher education has fallen sufficiently. Employers will to a lesser degree be able to screen candidates by using their educational credentials, and demand for other signals will increase.

Finally, there are some limitations to our study. We would note that the cognitive ability test used at conscription has remained essentially unchanged since the test was developed in the early 1950s, which means that test items may have become outdated and function differently due to changes in social context (e.g., the introduction of calculators in school would give pupils less training in pen-and-pencil calculations required for the conscription test). In addition, we only had cognitive ability scores available for men, which does not allow us to say anything about the correlation for women. Although research has shown that intelligence does not relate differently to school success for boys and girls^24^, and we would hence expect to observe similar patterns for women, this should be tested explicitly in future research.

## Materials and methods

### Data

We use information on male cognitive ability, educational attainment and taxable earnings in administrative register data from Statistics Norway. The data cover the male Norwegian birth cohorts 1950–1991, and contain a pseudonymous individual-level identifier that allows information to be linked across registers. The project was approved by the Norwegian Data Protection Authority (Datatilsynet). The Norwegian Center for Research Data and the University of Oslo's Data Protection Official have approved the Data Protection Impact Assessment for the project.

The cognitive ability data are in the form of stanine scores from compulsory military conscription testing, available for the large majority of all males who are typically scored around age 18/19. The stanine scores reflect performance on three timed tests covering arithmetic (30 items), word similarities (54 items) and figures (36 items). The test items were unchanged throughout the period, but the scoring norm was changed for the 1961 and later cohorts to accommodate the Flynn effect. Additionally, the mathematics part of the test was changed to a multiple-choice format around the 1980 test year.

Information on education comes from the educational registry. Educational attainment is defined in four separate ways, to assess robustness of results across measures: (1) years of schooling at the age of 30, (2) the “global” or constant educational-earnings rank (calculated from pooled birth cohorts), (3) the cohort-specific or variable educational-earnings rank, and (4) latent educational liability that treats observed education as binned ranges of an underlying continuous liability. The second and third measures explicitly consider the economic benefits of specific educational credentials, while the last measure adjusts for the “mechanical” measurement error in correlation measures when using an ordinal outcome variable.

Measurement of earnings rank draws on annual earnings, which are available since 1967. The algorithm calculating lifetime rank first computes the individual’s percentile position in the male birth cohort earnings distribution at each age between ages 30 and 60 (or the highest observed age for those born after 1961) and uses the average of age-specific percentiles to form the individual’s lifetime percentile rank. The constant educational-earnings rank measure is computed as the average lifetime rank in four-digit educational attainment cells, using the pooled 1930 through 1981 birth cohorts. For the time-varying educational-earnings rank measure, we compute the average rank in each four-digit attainment cell using individuals born 10 years earlier than those in our sample (i.e., matching attainment of those born 1950–1991 with earnings rank of those born 1940–1981).

Figures S1 and S2 (in the Supplement) show descriptive statistics for all but one measurement across birth cohorts (not feasible for educational liability measures) as well as the size of each of these cohorts. The statistics show fluctuations and trends in line with the extant literature, e.g. the emergence and reversal of the Flynn effect and general educational upgrading over cohorts. The correlations reported should not be affected by fluctuations in the variance and mean of our measures, as correlations are standardized within each birth cohort, ensuring comparability across time.

### Statistical methods

Correlations between cognitive ability and educational attainment are calculated for each of the birth cohorts separately using standard Pearson product-moment correlations. In addition, as the years of education show substantial bunching and a decidedly non-normal distribution, we also calculate polychoric correlations that model years of education as reflecting binned ranges of an underlying latent educational liability—where the latent thresholds separating different attainment categories is allowed to vary across birth cohorts. 95% confidence intervals are calculated for all four series.

### Supplementary Information

Supplementary Information.

## Data Availability

Norwegian privacy regulations limit our ability to share our register data, but we can provide guidance on how to request access to these data. The leading author can be contacted for more information on how to apply for the data.
