# Supplementary material for: Correlation between cognitive ability and educational attainment weakens over birth cohorts
Source: Sci Rep. 2023 Oct 18;13:17747. doi: 10.1038/s41598-023-44605-6 (PMC10584829; doi:10.1038/s41598-023-44605-6)
Supplement: Supplementary file 1 — Supplementary Information. [file 41598_2023_44605_MOESM1_ESM.docx]

**Supplementary material:**

**Correlation between cognitive ability and educational attainment weakens over birth cohorts**

**Summary statistics for the variables of interest**

**
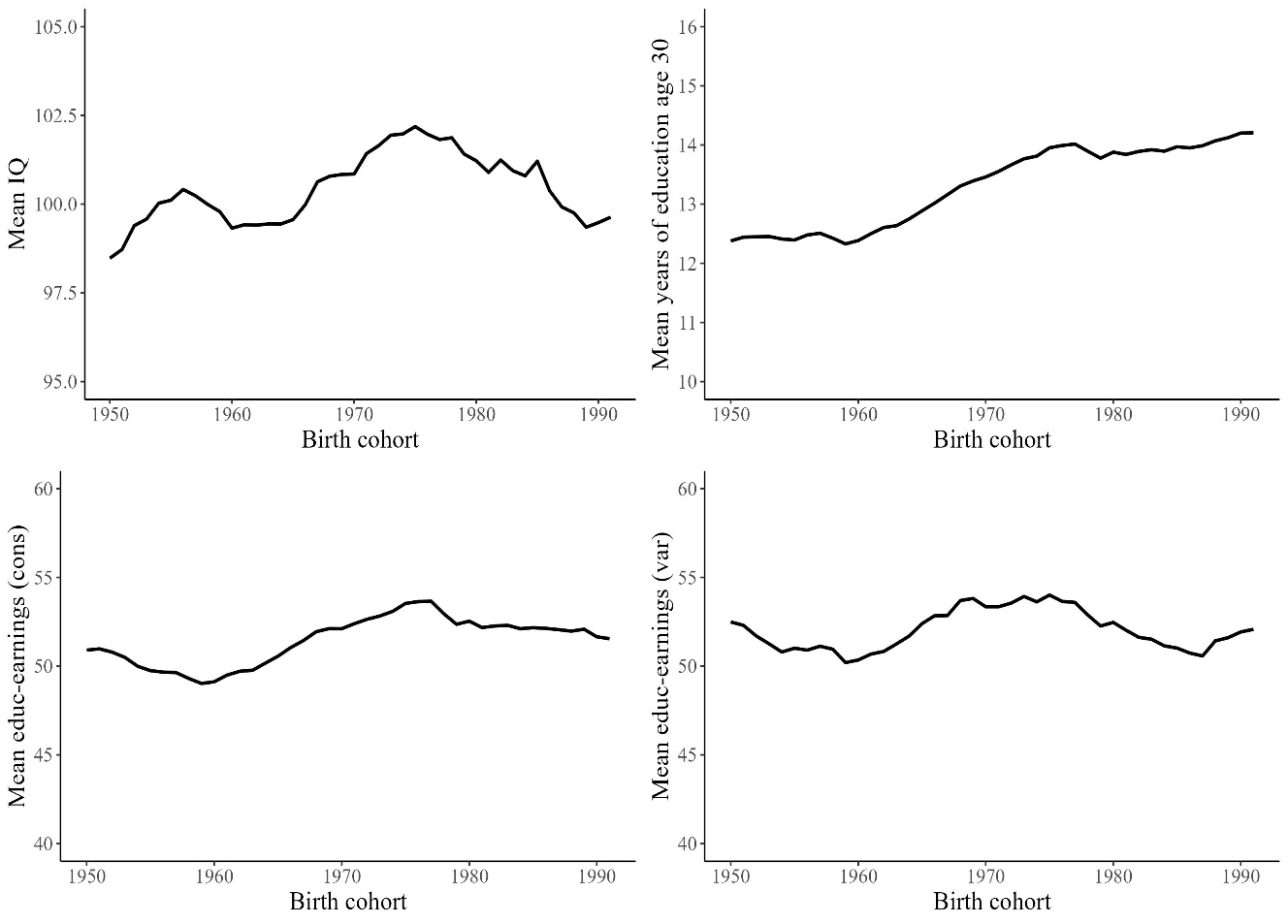
**

**Figure S1.** Trends in the mean values of the main variables of interest

**
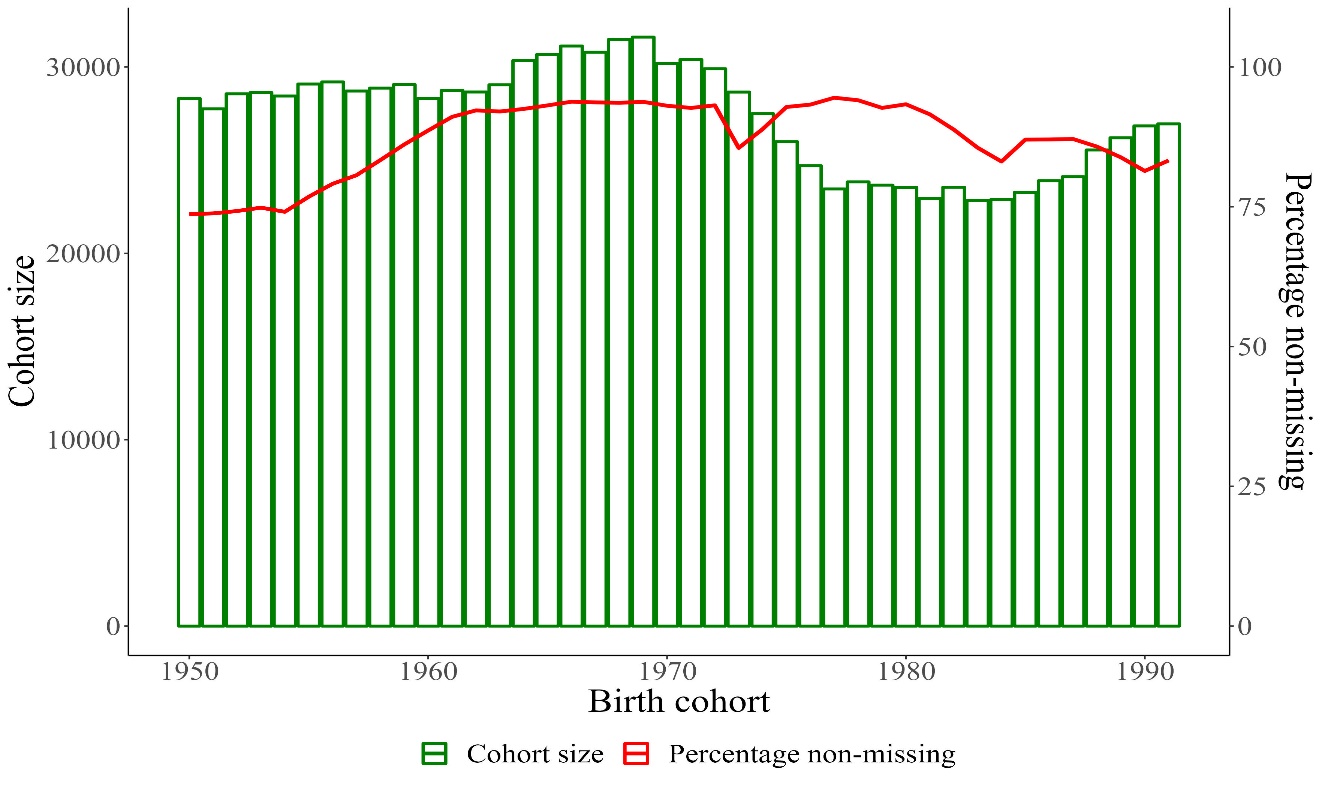
**

**Figure S2.** Cohort size and percentage of non-missing cases across birth cohorts

**Robustness check: Correlation between cognitive ability and education after age 19**

**
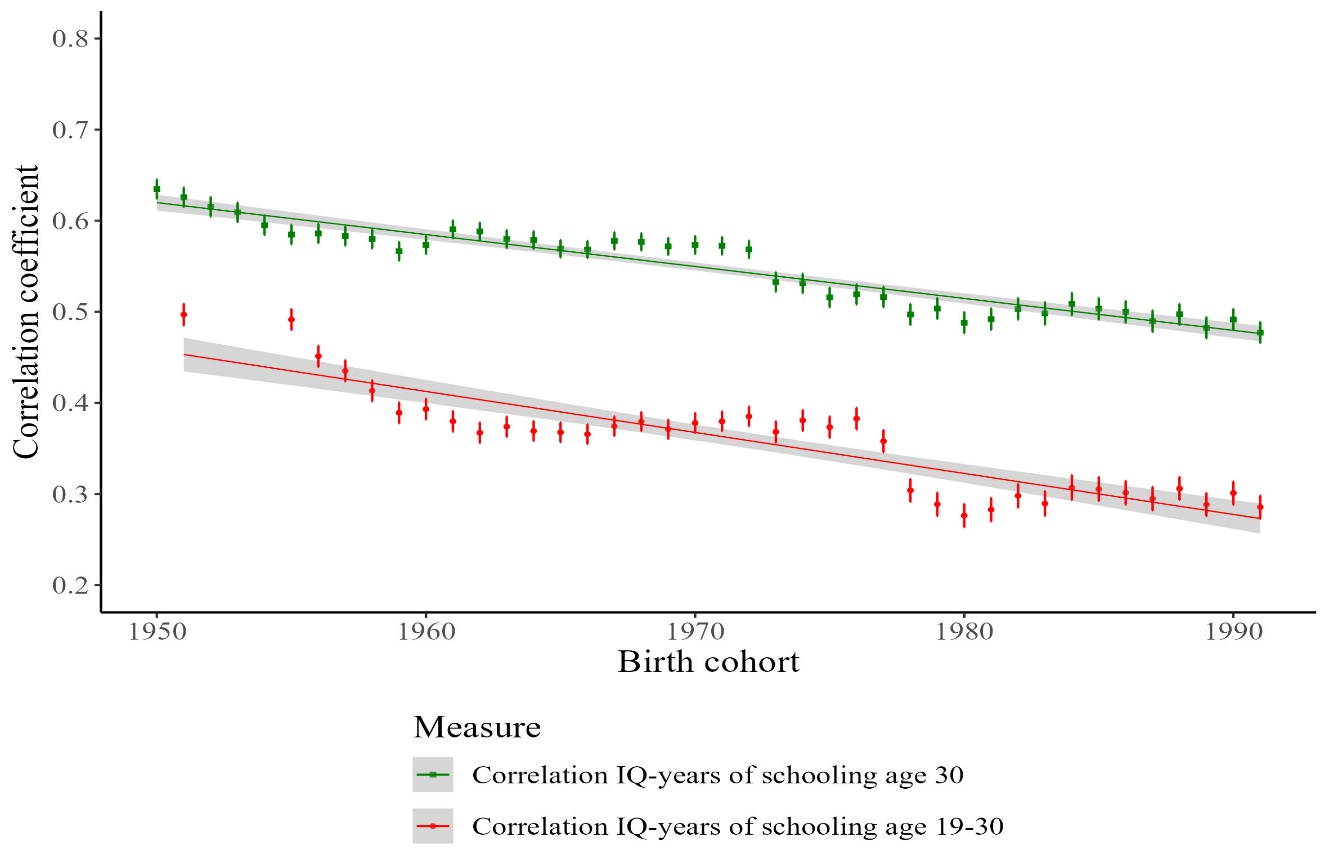
**

**Figure S3.** Comparing the correlation across birth cohorts between cognitive ability and education at age 30 to education achieved between age 19 and 30 (95 % confidence intervals of trend in grey)

*Note*: Because the education data draws on the 1970 census and the national education database established in 1974, educational attainment at age 19 is missing for birth cohorts 1950, 1952, 1953, and 1954.

**Robustness check: Predicting educational attainment by cognitive ability, regression models without and with control for father earnings rank**

**
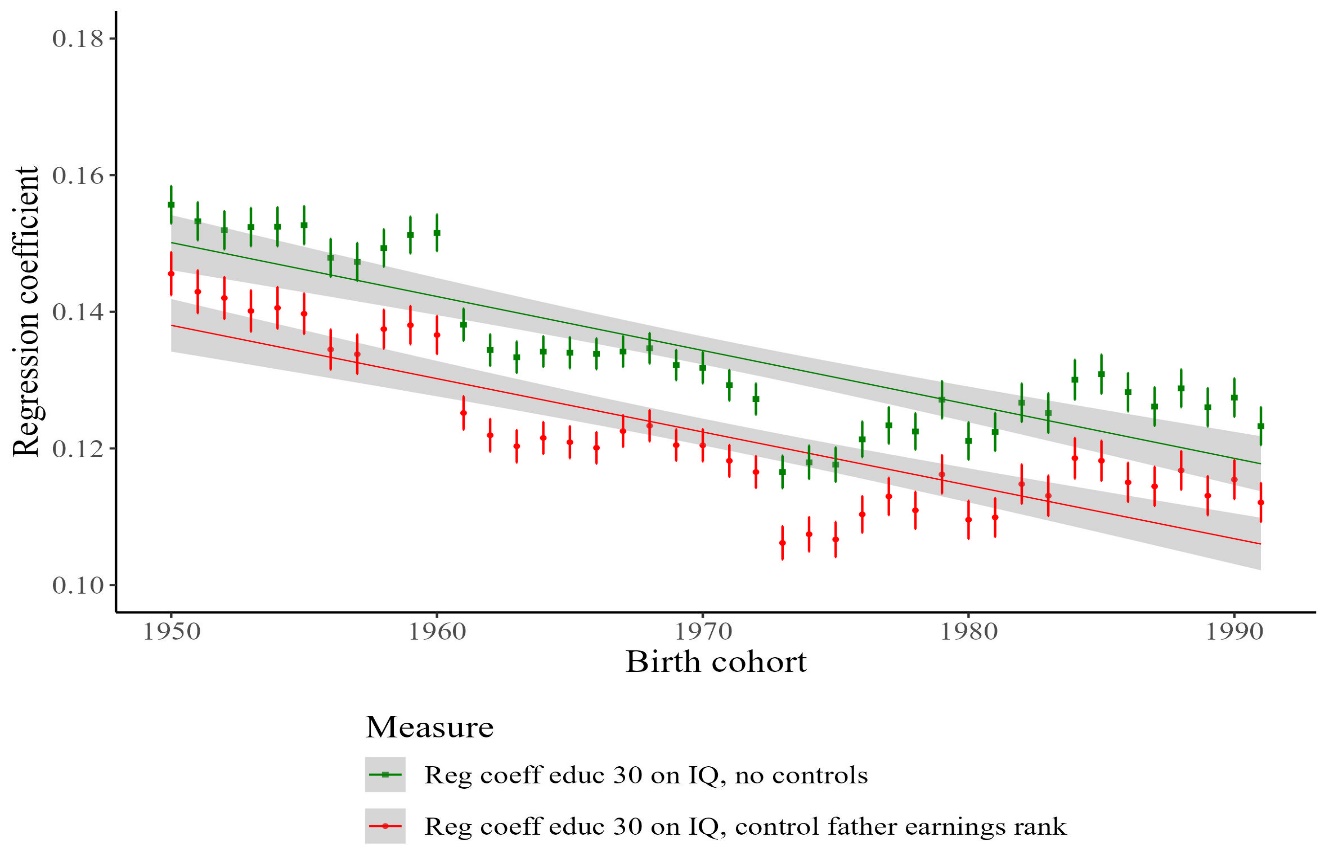
**

**Figure S4.** Comparing the regression coefficient across birth cohorts of cognitive ability predicting education at age 30, with and without control for father’s earnings ranks (95 % confidence intervals of trend in grey)

*Note*: Father’s earnings rank is computed from annual earnings data (available since 1967) and gives the father’s lifetime earnings rank relative to males born the same year
